# Supplementary material for: Characterization of New Small-Spored Alternaria Species Isolated from Solanaceae in Algeria
Source: Life (Basel). 2021 Nov 24;11(12):1291. doi: 10.3390/life11121291 (PMC8704928; doi:10.3390/life11121291)
Supplement: Supplementary file 1 [file life-11-01291-s001.zip › life-1466153-supplementary.pdf]

Supplementary Materials

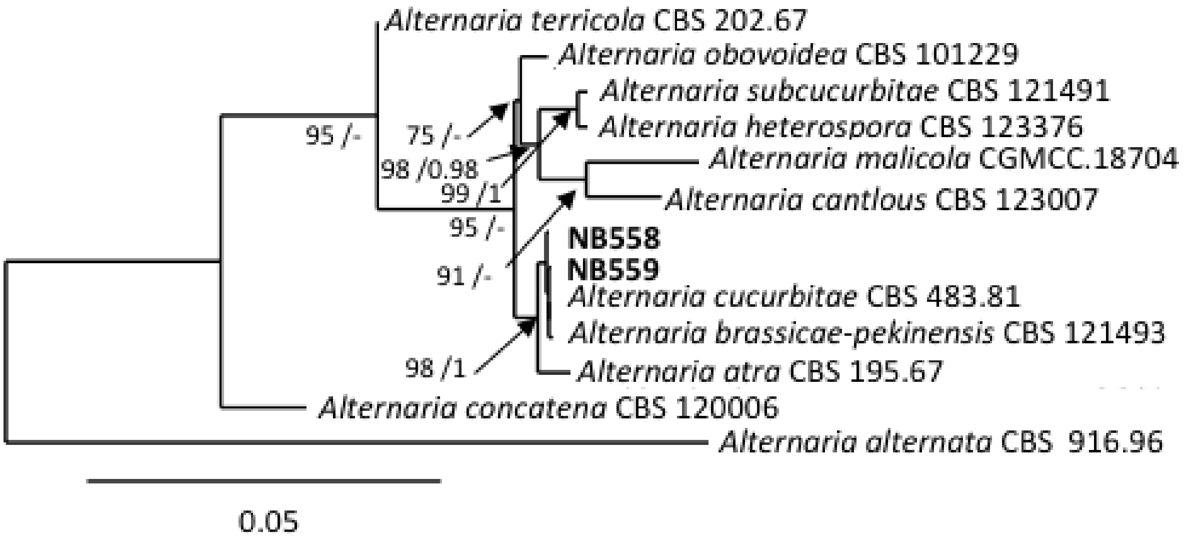

Figure S1. Phylogenetic tree reconstructed by the maximum likelihood method from the alignment of *gpd, tef1, rpb2, Alt a1* and *MAT1-2-1* of *Alternaria* isolates from section *Ulocladioides* for which these sequences are available. Bootstrap support values greater than 75% and Bayesian posterior probabilities greater than 0.95 are indicated near nodes. The GenBank acc. no. of sequences of the strains included as references were retrieved from [1].

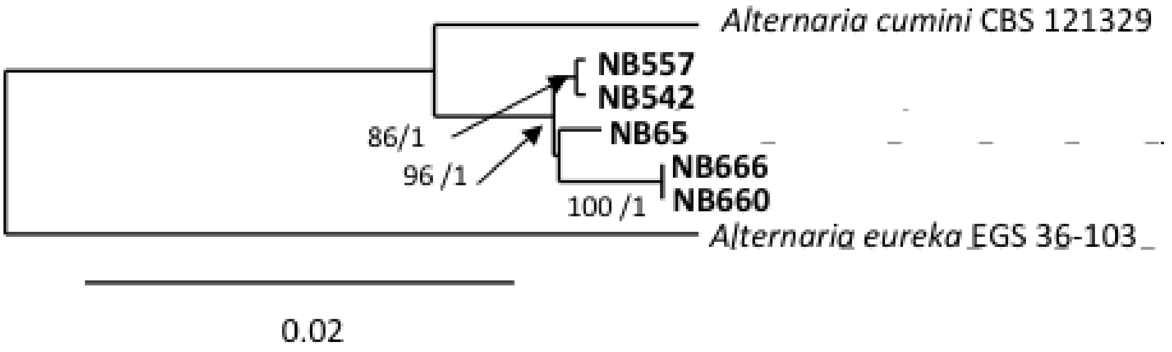

Figure S2: Phylogenetic tree reconstructed by the maximum likelihood method from the alignment of *ATPase, Act, Alt a1* of *Alternaria* isolates from section *Eureka.* Bootstrap support values greater than 75% and Bayesian posterior probabilities greater than 0.95 are indicated near nodes. The GenBank acc. nos. of sequences of the *Alternaria eureka* strain were retrieved from [2]. Acc. nos. of *Act* for *A. cuminicola* sp. nov. isolates are:. MK940320 (NB65), OK35889 (NB542), OK358898 (NB557), OK358899 (NB660), OK358900 (NB666). The nucleotide sequences of *ATPase, Act* and *Alt a1* for the type strain of *Alternaria cumini* (CBS 121329) were obtained during the present study and are referred in GenBank under the following acc. nos.: OK358913, OK358901 and OK358906, respectively.

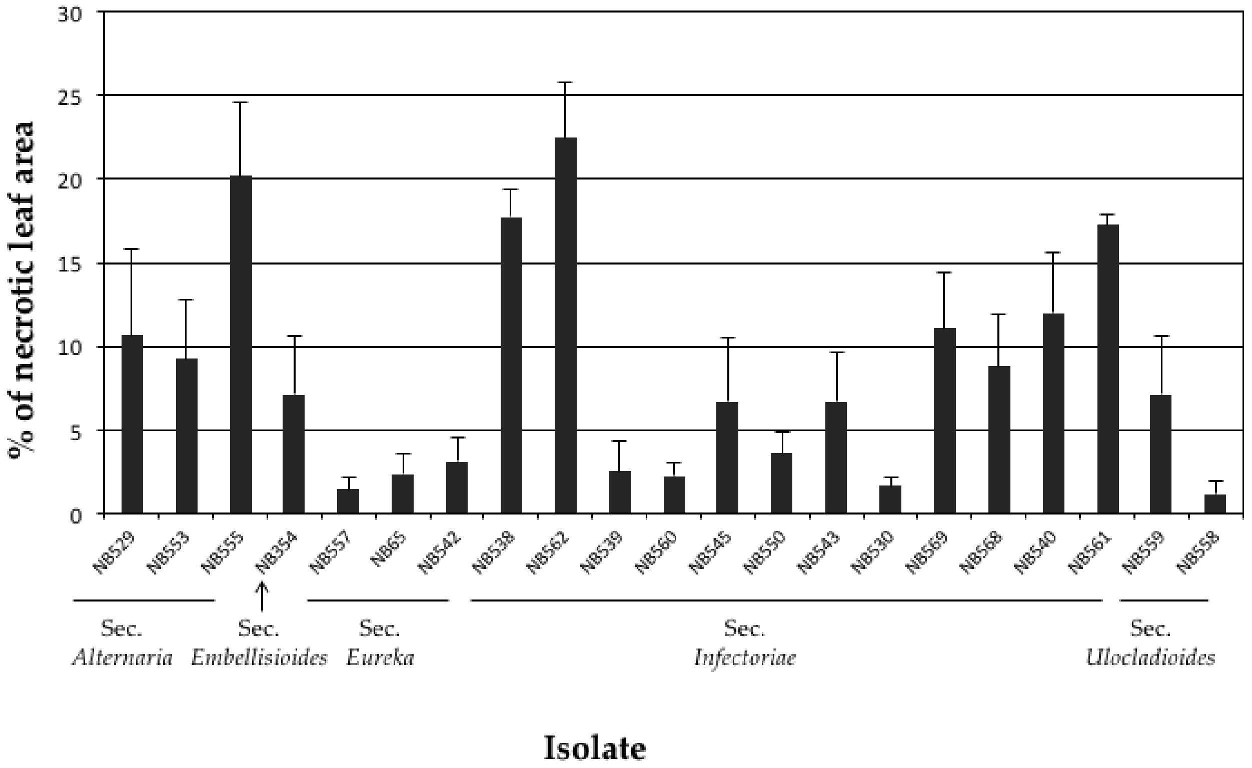

Figure S3. Pathogenicity testing of *Alternaria* isolates on *Solanum tuberosum* var. Désirée. Percentage of leaf necrotic area recorded at 21 DAI on plants inoculated with 18 *Alternaria* isolates plus 3 strains from section *Alternaria* (NB529, NB553, NB555).

Reference

1. Dang, J.L.; Gleason, M.L.; Li, L.N.; Wang, C.; Niu, C.K.; Zhang, R.; Sun, G.Y. *Alternaria malicola* sp. nov., a New Pathogen Causing Fruit Spot on Apple in China. *Plant Dis.* **2018**, *102*, 1273–1282.
2. Lawrence, D.P.; Gannibal, P.B.; Peever, T.L.; Pryor, B.M. The sections of *Alternaria*: Formalizing species-group concepts. *Mycologia* **2013**, *105*, 530–546. https://doi.org/10.3852/12-249.
